# Spirometry profiles among pregnant and non-pregnant African women: a cross-sectional study

**DOI:** 10.1186/s12905-022-02081-6

**Published:** 2022-12-02

**Authors:** Jacktan Josephat Ruhighira, Fredirick L. Mashili, Alexander Mtemi Tungu, Simon Mamuya

**Affiliations:** 1 Department of Physiology, University of Dodoma, P. O Box 395, Dodoma, Tanzania; 2 Department of Physiology, Muhimbili University of Health and Allied Sciences, P. O Box 65001, Dar es Salaam, Tanzania; 3 Department of Environmental and Occupational Health, Muhimbili University of Health and Allied Sciences, P. O Box 65001, Dar es Salaam, Tanzania

**Keywords:** Spirometry, Pregnancy, Women, Africa, Lung, Respiratory, FVC, FEV, PEF

## Abstract

**Background:**

Spirometry is a commonly used lung function test. It assesses respiratory functions by measuring the air volume and the rate at which a person can exhale from lungs filled to their total capacity. The most helpful spirometry parameters are: forced vital capacity (FVC), forced expiratory volume in one second (FEV1), and peak expiratory flow (PEF). Pregnancy derives an altered physiological state due to hormonal and anatomical changes that affect the respiratory system. Despite that, spirometry is less commonly done during pregnancy, and if done, test results are evaluated against non-pregnancy references.

**Objective:**

This study aimed to explore spirometry profiles in pregnant and non-pregnant women and describe their differences.

**Methodology:**

This cross-sectional study involved age-matched pregnant and non-pregnant participants recruited from Mnazi Moja ANC and Muhimbili University (MUHAS). A digital spirometer was used to assess respiratory function. Data were entered and analyzed using SPSS version 23. The mean spirometry values of pregnant participants were compared to those of non-pregnant participants using an independent sample t-test. A *p*-value of < 0.05 was considered statistically significant.

**Results:**

The study included 92 pregnant and 98 non-pregnant participants subjected to spirometry. Both FVC and FEV1 values were significantly lower in pregnant than in non-pregnant participants (2.7 ± 0.5 L vs. 2.9 ± 0.5 L; *p* < 0.01 and 2.2 ± 0.4 L vs. 2.5 ± 0.4 L; *p* < 0.01 respectively). In addition, pregnant participants had significantly lower mean PEF values than their non-pregnant counterparts (303 ± 84 L/min versus 353 ± 64 L/min; *p* < 0.01).

**Conclusion:**

Spirometry test values are lower in pregnancy than in non-pregnant participants.

**Recommendations:**

Interpreting the spirometry test values of pregnant women using references obtained from non-pregnant women may be inappropriate. Future studies should evaluate the appropriateness of predicting spirometry values of pregnant women using reference equations derived from non-pregnant women.

## Background

Lung function tests are investigations done to assess the ability of the lungs to exchange gasses and possible mechanical deterioration of the lungs, respiratory muscles, and chest wall [1]. They include spirometry, lung volumes test, lung diffusion capacity, pulse-oximetry, arterial blood gas analysis, and the fraction of exhaled nitric oxide test [1]. Spirometry is the most commonly used lung function test [1]. It assesses the functions of the lung tissue, chest wall, respiratory muscles, and airways by measuring the air volume and the rate at which a person can exhale from the lungs filled to their total capacity [2]. The most helpful spirometry parameters are: forced vital capacity (FVC), forced expiratory volume in one second (FEV1), and peak expiratory flow (PEF) [3]. FVC is the maximum air volume a person exhales forcefully after inhaling maximally. FEV1 is the volume of air exhaled in the first second of FVC measurement. PEF is a person's maximum rate at which a person forcefully exhales after maximal inhalation.

Spirometry parameters vary depending on age, sex, height, weight, body position, and race or ethnic group [4]. Most parameters peak at 20–25 years before they start to decline [5]. The most affected parameters are FVC and FEV1 [5]_._ These parameters differ between males and females, mainly due to biological and body size differences [6, 7]. Also, they differ between the known races of the world [7] and vary when taken in different positions (sitting, standing, or laying) [8]. The influence of age, sex, body size, race, and positions are related to expiratory muscle mass and strength, chest wall compliance, airway resistance, and lung tissue elasticity [5–11]. A growing body of evidence shows that pregnancy influences expiratory muscle mass and strength, chest wall compliance, airway resistance, and lung tissue elasticity [12–15].

Even though pregnancy is not a disease, it derives an altered physiological state primarily due to accompanied hormonal changes [16]. Progesterone and estrogen are pregnancy-induced physiological changes' primary triggers and drivers [17–19]. Growing gravid mechanically interferes with lungs and respiration. The diaphragm and lungs are displaced upward [12], and the ribcage volume [13] and chest wall compliance decrease [14] with uterine growth. Respiratory muscles respond to growing abdominal volume by increasing their separation breadth, stretch, and insertion angle [15]. These changes often cause nocturnal dyspnea, chest discomfort, and difficulty breathing, especially during late pregnancy [20].

Respiratory conditions are common in pregnancy [21], and restrictive disorders, if they occur during pregnancy, severely affect  the lung function profile [14] more than the general population [22]. Respiratory conditions are associated with adverse perinatal outcomes such as prematurity, small neonates for gestational age, and admissions to intensive care units [23–25]. Despite that, spirometry is less frequently performed among pregnant women, even those with conditions that affect respiratory function [26]. There is a lack of reference values for spirometry parameters and their associated factors during pregnancy in Sub-Saharan Africa, including Tanzania. Few studies available only involved non-pregnant African participants [27–29]. The use of spirometry references obtained from non-pregnant women is likely to underestimate the test values of pregnant women. Therefore, evaluating the spirometry profile among the pregnant population was substantial.

### Objectives and hypothesis

The general objective of this study was to explore the spirometry profiles of pregnant and non-pregnant women. Specifically, this study aimed to describe the spirometry profiles and their affecting factors among non-pregnant and 6–36 weeks pregnant women and assess their differences. We hypothesized that age, height, weight, parity, and gestational age affected spirometry profiles. We also thought that pregnant and non-pregnant women's spirometry profiles differed.

## Methodology

### Study design and participants

This cross-sectional study was conducted in Dar es Salaam, Tanzania, from May to July 2021. Dar es Salaam is the largest city and industrial center of Tanzania, eastern Africa. The city's projected population was 5,401,814 by 2020 [30]. The study involved pregnant participants attending antenatal clinic services at Mnazi Mmoja hospital and non-pregnant participants recruited from among female persons at Muhimbili University (MUHAS). Mnazi Mmoja Hospital, located 1.2 km from the city center, has a reproductive health center serving more than 100 women daily. MUHAS is a public university accredited by the Tanzania Commission of Universities. The university's main campus, where non-pregnant participants were recruited, is about 2.7 km from the city center. The university had 3861 students and other non-student persons during the study period.

### Sample size

We used the formula for cross-section studies with quantitative variables published by Charan and Biswas [31] review to calculate the sample size.

$${\text{Sample size}} = {\text{Z}}_{{1 - \upalpha /2}}^{2} {\text{SD}}^{2} /{\text{d}}^{2}$$

where Z_1−α/2_ is a standard normal variate for a given level of significance (*p-*value); SD is a standard deviation for a variable obtainable from the previous or pilot study; d is an acceptable margin of error set by a researcher.

This study used a standard deviation (7.36) for the mean FEV1/FVC ratio in the second trimester from the previous study [32], the level of significance (*p*-value) of probability < 0.05, and the marginal error set to 1.6. We adjusted the sample size to 10% non-response. The sample size calculated for pregnant participants was 91, and we aimed to recruit a similar number of non-pregnant participants. We exceeded the computed sample size whereby this study involved 92 pregnant and 98 non-pregnant participants.

### Sample selection

A simple random sampling technique was employed to obtain pregnant participants. Upon consenting to participate, those meeting all the criteria were assigned numbers. Then potential pregnant participants were selected using a table of random numbers. All eligible participants at MUHAS who were not pregnant were invited to participate in this study. Convenient sampling was used to obtain non-pregnant participants to match pregnant participants' group characteristics as much as possible.

### Eligibility and inclusion criteria

Included in this study were African decency pregnant women of age 18–35 years and gestational ages from 6 to 36 weeks. The study did not include pregnant women below 18 as their presumed immature reproductive system could influence the observed spirometry profile. Meanwhile, women over 35 years were not included as they are likely to experience pregnancy-related complications in advanced maternal age [33–35]. The first five weeks were not included due to the difficulty of diagnosing pregnancy at this gestational age [36]. We did not include the term pregnancy due to safety issues related to increased intra-abdominal pressure during the spirometry maneuver [37]. Since first-visit weight was to be used to calculate BMI in pregnancy instead of pre-pregnancy weight, only women who booked their first visit in their first trimester were included. Non-pregnant participants were recruited if they had similar criteria except for pregnancy.

### Exclusion criteria

Participants among whom spirometry is contraindicated [38] were excluded from the study. Screening for contraindications was done on every potential participant before enrollment. Also, participants already known to have any lung disease or any other diseases affecting lung function or exposed to tuberculosis in the past year, had a lax uterus or a history of mid-trimester abortion [38], had a history of smoking, had multiple pregnancies, or failed to obtain any acceptable and repeatable spirometry measurement were excluded. Measures were regarded as repeatable if they didn't deviate by more than 150 ml [3]. Participants who were pregnant in the last 42 days before the data collection weren't involved to exclude the effects of previous pregnancy.

### Variables

Independent variables were age, pregnancy status, parity, gestational age, height, and weight. Age was defined as the period passed since birth and measured in years as reported by subjects. Pregnancy status was defined as the presence or absence of  a detectable pregnancy. Gestational age was determined as to how long a woman has been pregnant, measured by the number of weeks calculated from the last menstruation date. This age was further categorized into corresponding trimesters, each covering 12 weeks. Parity was defined as how many times a woman had given birth to a child, whether deceased or alive, after being pregnant for at least 28 weeks, measured by subject reporting. Height was defined as vertical distance measured in centimeters from the lowest point of the body to the highest in a straight upright position. Weight was defined as the quantity of mass of a body measured in kilograms. Height and on-date weight for non-pregnant or on the first visit for pregnant participants obtained from the antenatal card was used to obtain body mass index (BMI).

Dependent variables were spirometry parameters: FVC, FEV1, PEF, and FEV1/FVC ratio. FVC was defined as the maximum air volume exhaled forcefully and rapidly following deep inhalation. FEV1 was defined as the volume of air exhaled in the first second of the FVC test. PEF was described as the speed at which a person exhaled after inhaling maximally.

### Data collection tool and data collection process

A structured checklist collected demographic information, anthropometry, and spirometry measurements. The tool was tested through a pilot test administered to 10 pregnant and nine non-pregnant participants. The interview was used to collect demographic, and pregnancy information using a structured checklist adapted from the maternal recall questionnaire [39]. The absence of pregnancy was confirmed among the non-pregnant group using a standard urine pregnancy test at the MUHAS physiology laboratory. Height was measured in an erect standing position using the SECA stadiometer. On-date weight was measured using the SECA adult weighing machine placed on a flat surface whereby participants stood looking straight ahead while hands were positioned at their sides [40]. Weight on the first visit for pregnant participants was obtained from antenatal cards. It was used to calculate body mass index (BMI) in pregnancy instead of pre-pregnancy weight, which was unavailable. BMI was further categorized into underweight (BMI < 18.5), average weight (BMI = 18.5–24.9), overweight (BMI = 25–29.9), and obesity (BMI ≥ 30).

The spirometry was done using a computerized EasyOne® Diagnostic spirometer in a sitting position with a nose clip. Test mode was set to DIAGNOSTIC, prediction reference was set to NHANES III, and select value was set to BEST VALUE. Participants were coached for correct maneuvers using protocols adapted from NHANES 2011 [41] and ATS and ERS [3] spirometry examination manuals. They were instructed to elevate the chin, straighten the neck, and then take a deep breath to fill the lungs. Then, a disposable mouthpiece was placed between the teeth and above the tongue before blowing up as fast and forcefully as possible until asked to stop after a minimum of six seconds. At least three acceptable and reproducible measurements were obtained, and the best values were recorded. Participants and personnel observed hand washing and stayed at least one meter apart without facing each other directly. Participants unwrapped and inserted the mouthpiece onto a properly sanitized spirometer on their own.

### Data management and analysis

Data was entered using version 23 of the SPSS software. The FVC, FEV1, and PEF were normally distributed and described using the mean, but FEV1/FVC was slightly skewed to the right; thus, the median was described. The one-way analysis of variance (ANOVA) was used to test the effect of factors such as age, weight, and height on FVC, FEV1, and PEF and Kruskal–Wallis for FEV1/FVC ratio. The difference between the mean of predicted and measured values within a person was analyzed using paired t-test. The mean spirometry values of pregnant participants were compared to those of non-pregnant participants using an independent sample t-test [42, 43]. Adjustments to potential confounders were made through the analysis of covariance (ANCOVA). A *p*-value less than 0.05 was considered significant.

### Ethical consideration

The study was cleared by MUHAS's ethical institutional review board. Permission to conduct the study was obtained from the Mnazi Mmoja hospital administration, local governments, and the MUHAS administration. All methods were in harmony with the Helsinki Declaration. Study protocols and objectives were revealed to participants. Written informed consent was prearranged and signed by participants before enrollment into the study. Pregnancy tests among the non-pregnant group were conducted privately at the MUHAS physiology laboratory. We did not collect personal identifying information. All other information gathered was used for research purposes only. Participants with abnormal measurements were recommended for medical evaluation as per Mnazi Mmoja hospital protocol.

Generally, spirometry is considered safe during pregnancy as no complications have been reported [38]. However, several safety precautions were taken to avoid potential complications related to the spirometry maneuver. Potential participants with any contraindication for spirometry [3, 38, 41] were excluded. Spirometry was done in seating as a precaution against possible lightheadedness due to exertion during the maneuver.

## Results

### Recruitment of participants

**Fig. 1:**
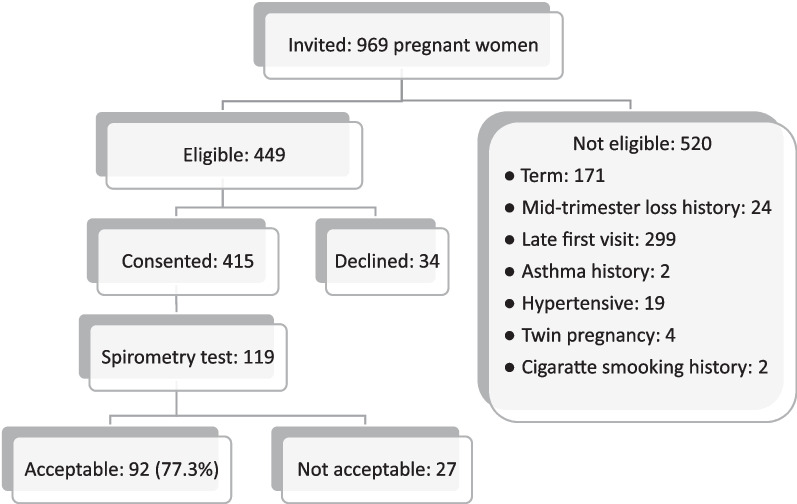
Schematic for recruitment of pregnant participants

All pregnant women who visited Mnazi Mmoja hospital during the study period were invited to participate in the study (Fig. 1). Among the eligible participants, 92 who produced acceptable and reproducible measurements were involved in this study. Then, out of 119 non-pregnant participants subjected to spirometry testing, 98 with acceptable and reproducible measurements were involved in this study.

### Description of characteristics of participants

The mean age of the study participants was 27 years (SD = 5). Their mean height was 157.4 cm (SD = 6.7), ranging from 135 to 173 cm tall. The mean weight was 67 kg (SD = 14.2), ranging from 47 to 117 kg. Among all participants, 46.7% were overweight or obese, 7.6% were underweight, and 52.4% had previously given birth at least once (Table 1).

**Table 1:** Characteristics of the participants (n = 190)

	Pregnant		Non-pregnant		*p*-value
	Frequency (%)	Mean ± SD	Frequency (%)	Mean ± SD	
*Age*					
18–19	2 (24)		7 (7.1)		
20–24	24 (25.5)		48 (48.7)		
25–29	25 (27.5)		20 (20.4)		
30–35	41 (44.6)		23 (23.7)		
Total	92	28 ± 5	98	26 ± 5	0.040
*Height*					
135–139	1 (1.1)		0		
140–149	11 (11.8)		11 (11.2)		
150–159	51 (55.3)		43 (43.9)		
160–169	29 (31.7)		40 (40.8)		
170–179	0		4 (4.1)		
Total	92	156 ± 6.4	98	158.5 ± 6.8	0.290
*Weight*					
41–50	6 (6.0)		20 (20.4)		
51–60	16 (17.5)		28 (28.6)		
61–70	24 (26.5)		24 (24.5)		
71–80	23 (24.4)		15 (15.3)		
81–90	16 (16.7)		8 (8.2)		
91–110	6 (6.3)		3 (3.1)		
Total	92	70.9 ± 13.8	98	62.6 ± 13.5	0.011
*BMI*					
Underweight	6 (6.4)		9 (8.7)		
Normal	37 (40.3)		50 (50.8)		
Overweight	30 (33.0)		16 (16.7)		
Obese	19 (20.3)		23 (23.7)		
*Parity*					
0	32 (34.8)		57 (58.2)		
1	32 (34.8)		16 (16.3)		
2	14 (15.2)		12 (12.2)		
3	12 (13)		6 (6.1)		
4	2 (2.2)		7 (7.1)		
Total	92		98		0.006
*Gestation age*					
First trimester	7 (7.6)				
Second trimester	31 (34.0)				
Third trimester	92	25.3 ± 7.9			

*p*-value for the characteristic differences between pregnant and non-pregnant participants

### Description of spirometry test values of participants

**Fig. 2:**
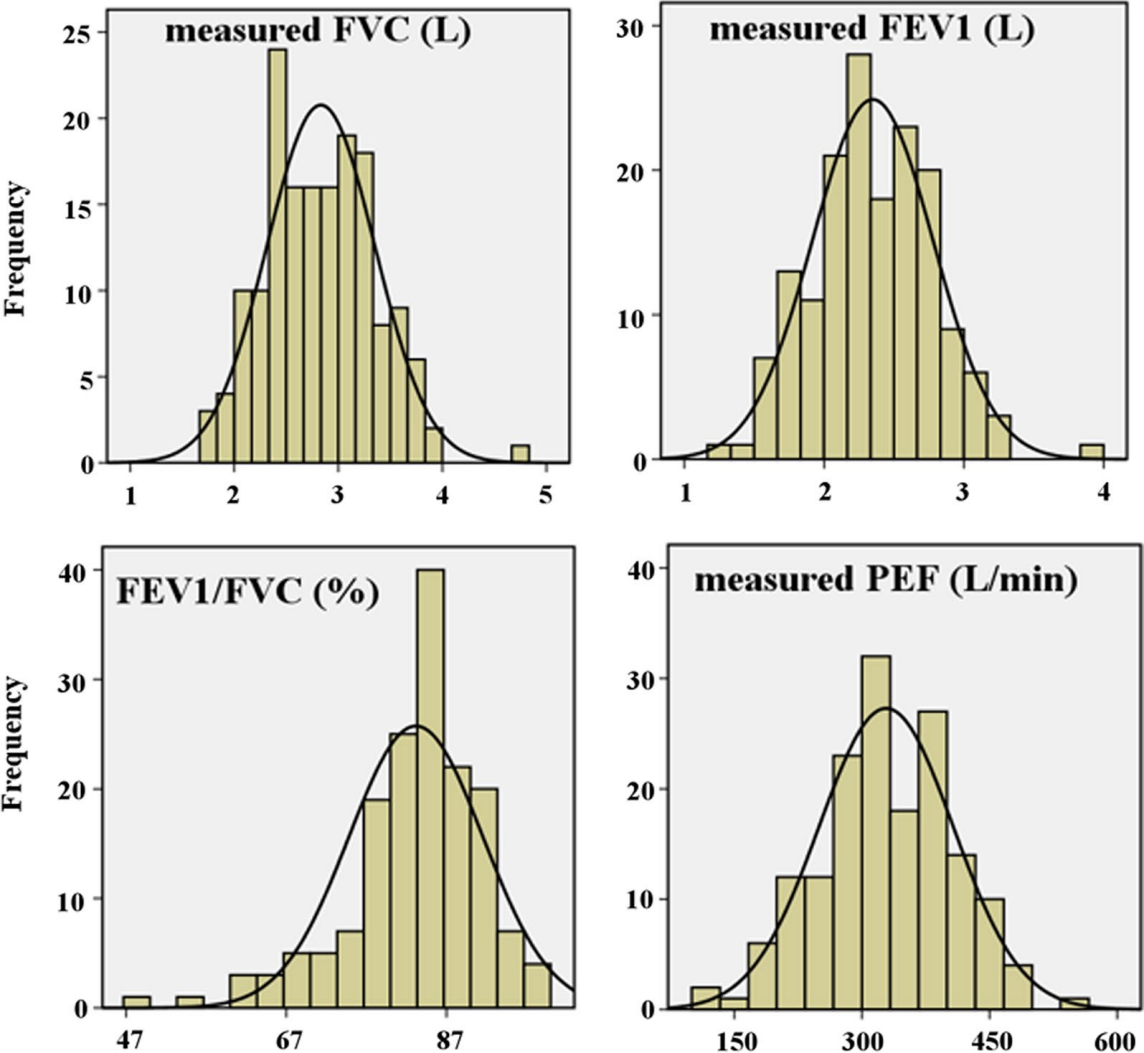
Histogram describing the distribution of spirometry test values of participants (n = 190)

The distribution of spirometry test values and their respective percentage predicted was normal except FEV1/FVC (in %) ratio (Fig. 2).

The mean FVC was 2.8 L (SD = 0.52) which was 94.9% (SD = 16.3) of the values predicted by age and height. Their mean FEV1 was 2.4 L (SD = 0.43) which was 90% (SD = 14.5) of predicted. The Median FEV1/FVC ratio was 84.3% (48.8–99.8). The mean PEF was 329.3 L/min (SD = 78.5) L/min which was 84.2% (SD = 19.8) of predicted (Table 2).

**Table 2:** Summary of the spirometry test values of the participants (n = 190)

	Mean ± SD		t value	*df*	*p*-value
	Pregnant	Non-pregnant			
*FVC (L)*					
Predicted	2.9 ± 0.28	3.1 ± 0.32			
Measured	2.7 ± 0.54	2.9 ± 0.48	− 3.041	189	0.006
FVC%	92.9 ± 18.6	96.3 ± 13.4	− 1.431	167	0.179
*FEVl (L)*					
Predicted	2.6 ± 0.22	2.7 ± 0.3			
Measured	2.2 ± 0.42	2.5 ± 0.41	− 4.512	189	0.000
FEV1%	86.9 ± 15.1	93.1 ± 13.4	− 2.990	189	0.003
*PEF (L/min)*					
Predicted	387.6 ± 23.6	397.9 ± 29.5			
Measured	303.2 ± 84.5	353.8 ± 63.6	− 4.647	169	0.000
PEF%	78.4 ± 21.8	89.7 ± 15.8	− 4.100	166	0.000
	Median (Range)				
FEVl/FVC (%)	83.7 (48.8–99.8)	85.1 (65.0–98.1)			0.281

*p*-value for the respective differences between pregnant and non-pregnant participants

### Factors affecting spirometry profiles

#### Age and spirometry test values

**Fig. 3:**
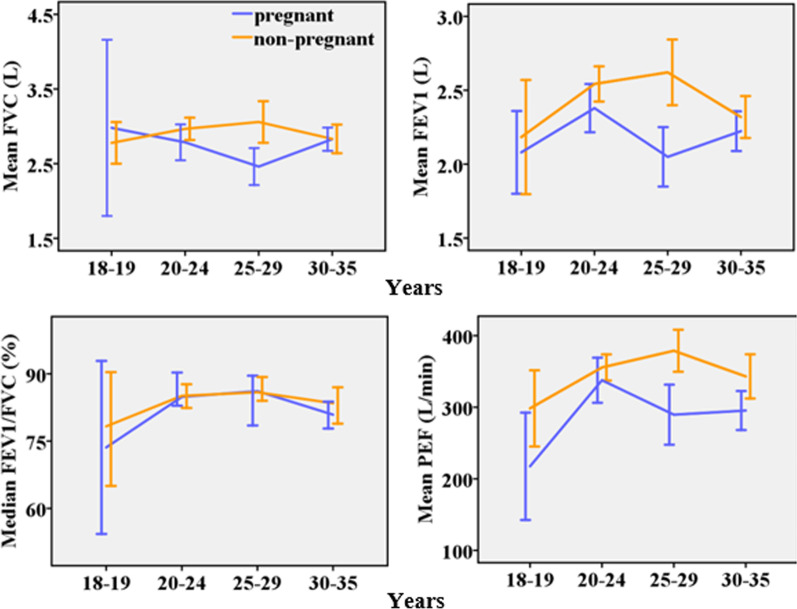
Plots of spirometry test values against age [n = 190, Error bar: ± 2SEM (95%CI)]

The relationship between age and spirometry profile appeared to be phasic, with an increase to peak then decrease (Fig. 3). The pattern was statistically significant among pregnant participants for FVC [F (3, 88) = 2.83; *p* = 0.043] and FVC% [F (3, 88) = 2.89; *p* = 0.04] even after adjusting for height but not after including weight in the ANCOVA. The pattern was statistically significant among non-pregnant participants for FVC [F (16, 81) = 2.44; *p* < 0.01], FVC% [F (16, 81) = 1, 79; *p* = 0.05], FEV1 [F (16, 81) = 2.53; *p* < 0.01], FEV1% [F (16, 81) = 1.81; *p* = 0.04], PEF [F (16, 81) = 2; *p* = 0.02], PEF% [F (16, 81) = 2.59; *p* < 0.01] and FEV1/FVC (*p* = 0.042) even after adjusting for height and weight.

#### Height and spirometry test values

**Fig. 4:**
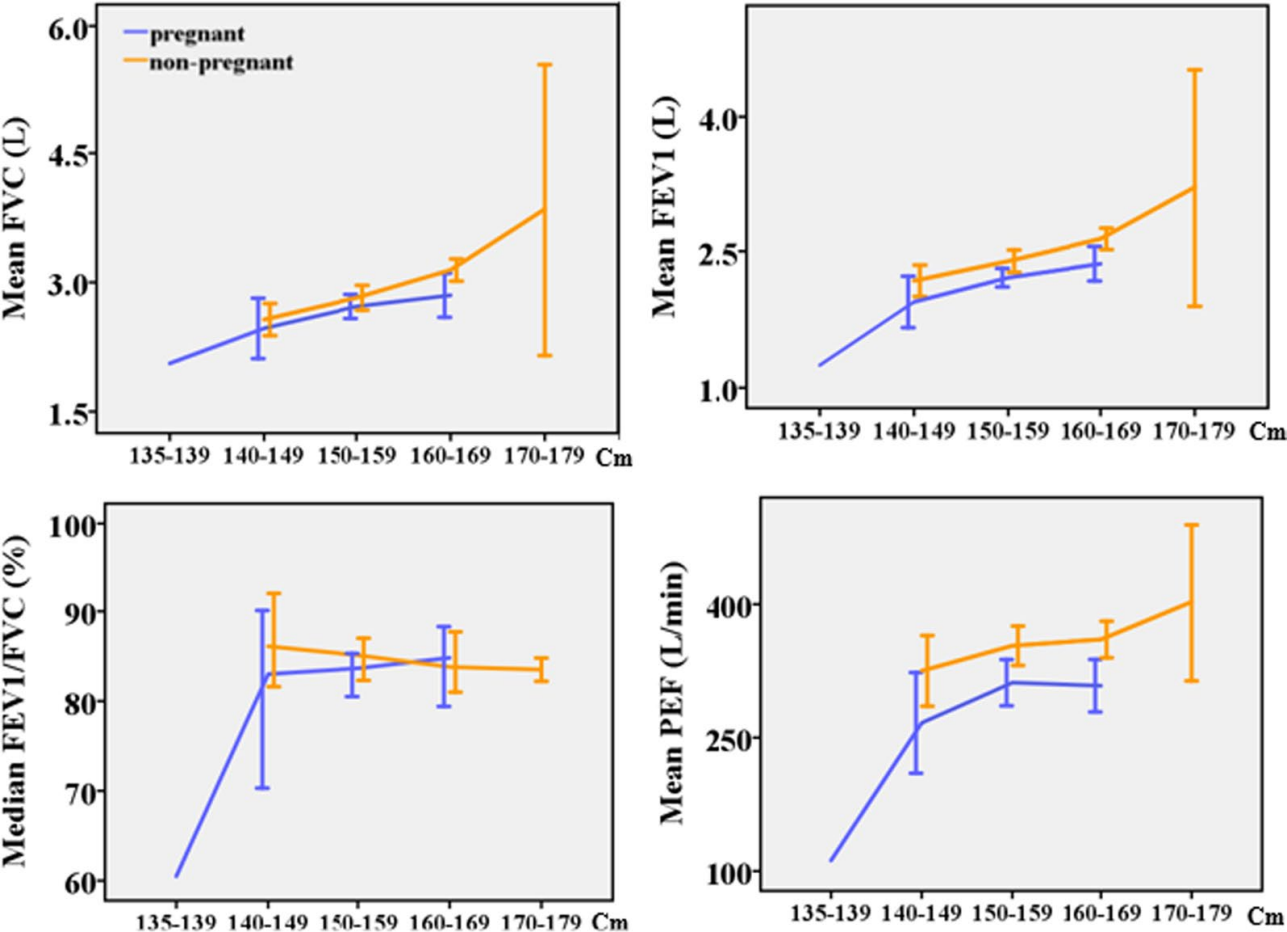
Plots of spirometry test values against height [n = 190, Error bar: ± 2SEM (95%CI)]

Spirometry values increased with height (Fig. 4) except for FVC%, FEV1%, and median FEV1/FVC. The pattern was statistically significant among pregnant participants for FVC [F (25, 66) = 1.88;* p* = 0.02], FEV1 [F (25, 66) = 2.54;* p* < 0.01], and PEF [F (25, 66) = 1.79;* p* = 0.03] but was no longer significant for FVC and PEF after adjusting for weight and age. The pattern was statistically significant among non-pregnant participants for FVC [F (3, 94) = 7.96; *p* < 0.01] and FEV1 [F (3, 94) = 6.65; *p* < 0.01] even after adjusting for age and weight.

#### Weight and spirometry test values

**Fig. 5:**
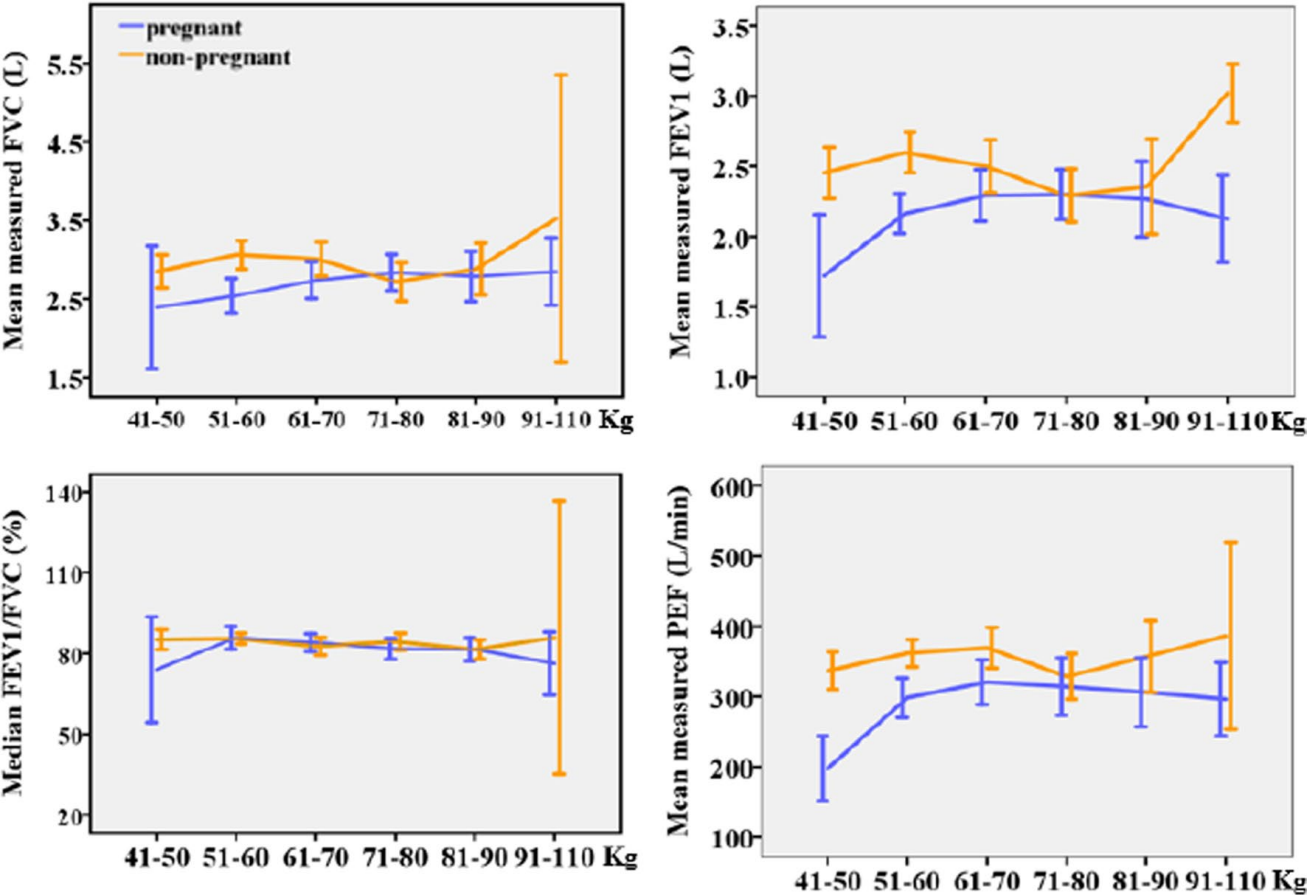
Plots of spirometry test values against weight [n = 190, Error bar: ± 2SEM (95%CI)]

Pregnant participants' mean FVC, FEV1, and PEF increased with weight until 60–70 kg, then decreased. The median FEV1/FVC of pregnant and non-pregnant participants remained unchanged as weight increased (Fig. 5). No pattern was statistically significant except for PEF (*p* = 0.010) of non-pregnant participants before adjusting for age and height.

**Fig. 6:**
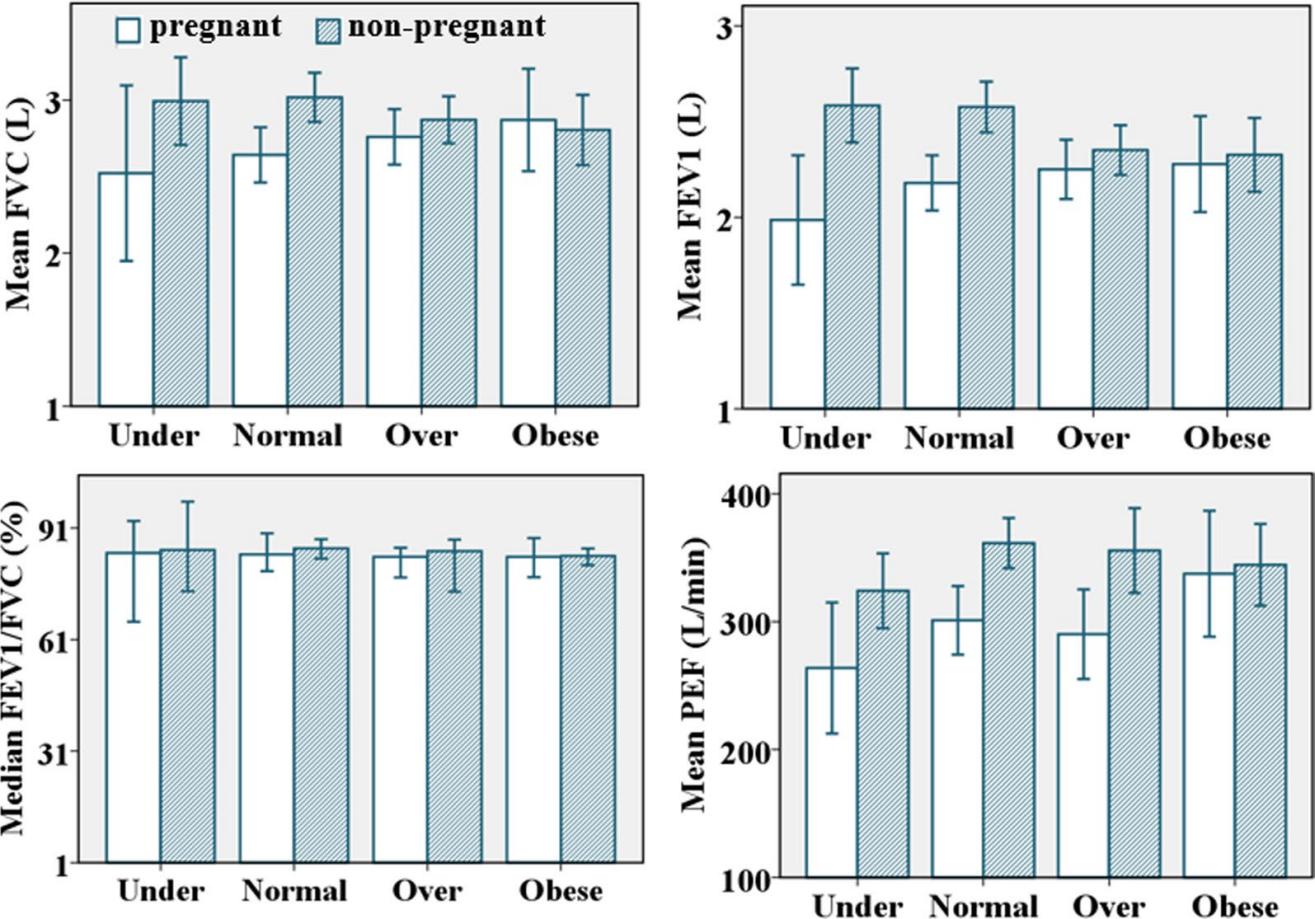
Plots of spirometry test values against BMI [n = 190, Error bar: ± 2SEM (95%CI)]

While mean spirometry test values of pregnant participants increased with BMI, mean values of non-pregnant participants decreased as BMI increased, and medians of the FEV1/FVC ratio remained relatively unchanged (Fig. 6). But the patterns were not statistically significant.

#### Parity and spirometry test values

**Fig. 7:**
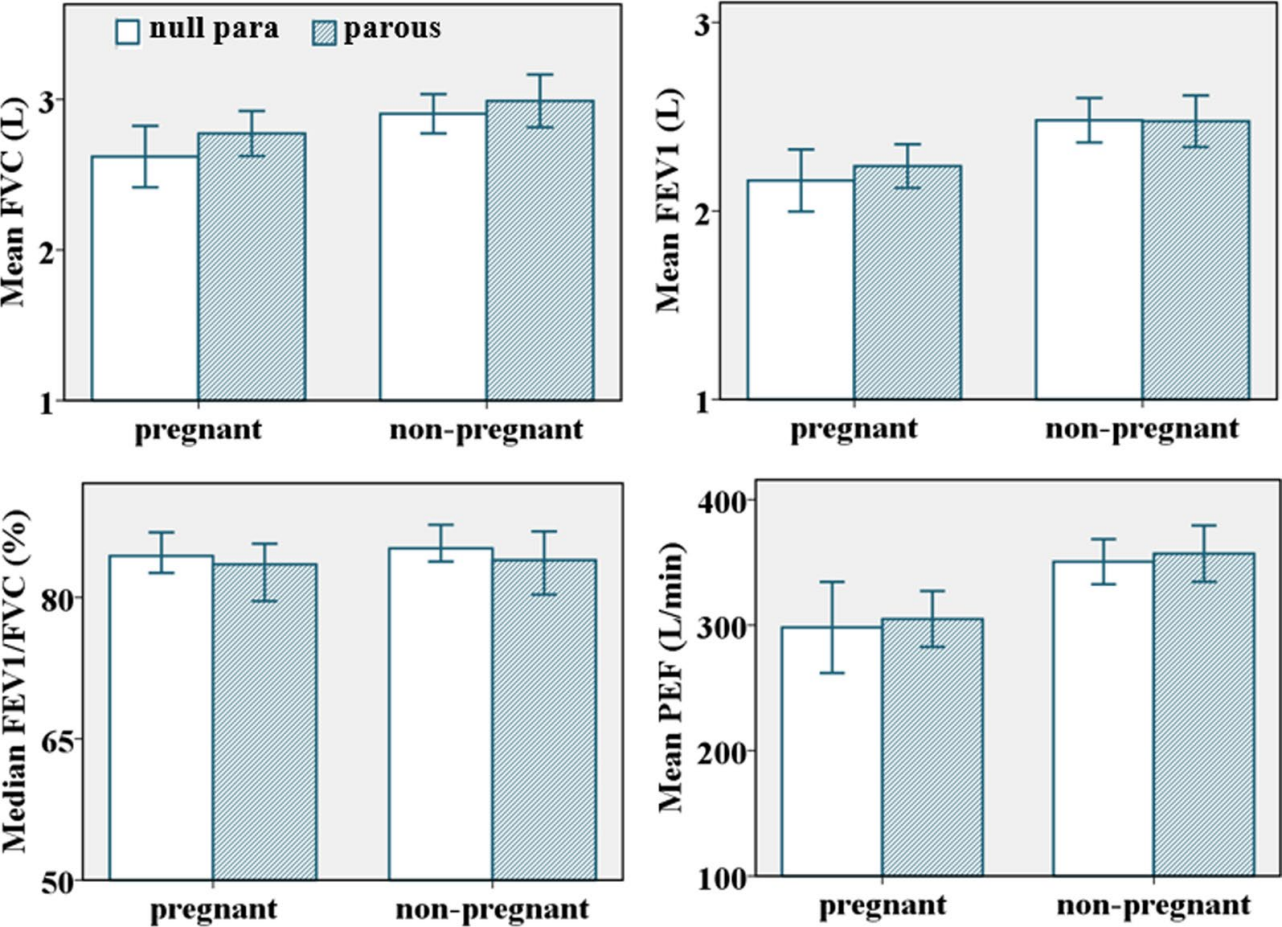
Plots of spirometry test values against parity [n = 190, Error bar: ± 2SEM (95%CI)]

The mean FVC, FEV1, and PEF of parous participants were higher than the mean of nulliparous participants. The median FEV1/FVC of nulliparous participants was higher than the parous participants (Fig. 7). However, the pattern was only statistically significant for median FEV1/FVC among pregnant participants (*p* = 0.035), and the mean of FVC% [F (4, 93) = 2.88;* p* = 0.03] and FEV1% [F (4, 93) = 3.89;* p* = 0.01] among non-pregnant participants even when adjusted for height but not when adjusted for age and weight.

#### Gestational age and spirometry test values

**Fig. 8:**
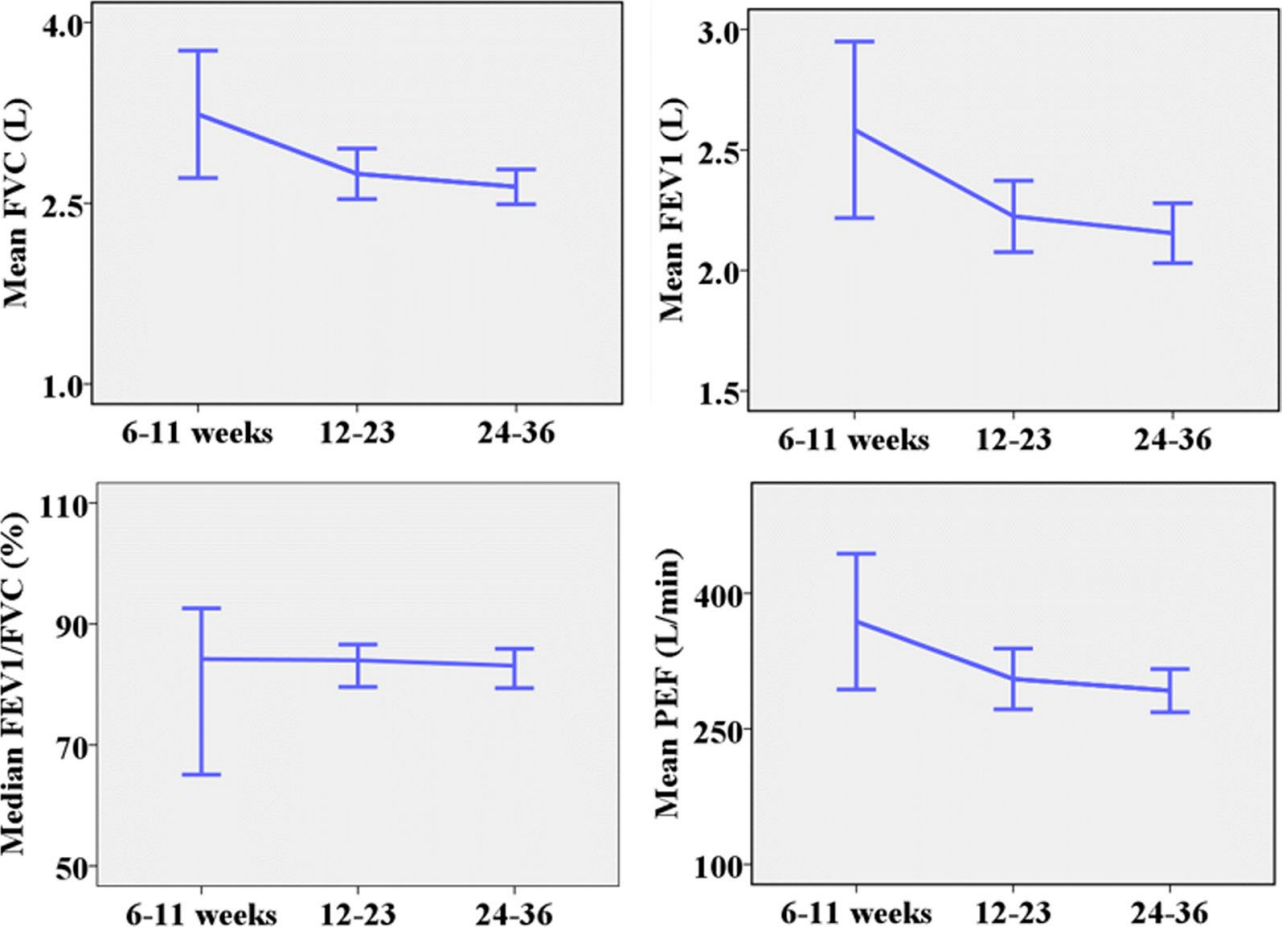
Plots of spirometry test values against gestational age [n = 92, Error bar: ± 2SEM (95%CI)]

The mean of FVC, FEV1, PEF, and their % predicted decreased as gestational age increased. The decrease was steeper from the first to the second trimester (Fig. 8). This pattern was statistically significant for FVC [F (2, 89) = 4.03;* p* = 0.02], FVC% [F (2, 89) = 6.81;* p* < 0.01], FEV1 [F (2, 89) = 3.15;* p* = 0.048] and FEV1% [F (2, 89) = 5.91;* p* < 0.01] even after adjusting for maternal age, height and weight.

### Difference between pregnant and non-pregnant participants' spirometry test values

The pregnant participants’ measured FVC [t (91) = − 3.97;* p* < 0.001], FEV1 [t (91) = − 8.39;* p* < 0.001] and PEF [t (91) = − 9.69;* p* < 0.001] as well as non-pregnant participants’ FVC [t (97) = − 2.86;* p* = 0.001], FEV1 [t (97) = − 5.17;* p* < 0.001] and PEF [t (97) = − 7.12;* p* < 0.001] were significantly lower than values predicted based on age and height.

Meanwhile, the pregnant participants’ mean FVC [t (189) = − 3.04;* p* = 0.006], FEV1 [t (189) = − 4.51;* p* < 0.01], FEV1% [t (189) = − 2.99;* p* = 0.003], PEF [t (169.5) = − 4.65;* p* < 0.001] and PEF% [t (165.9) = − 4.1;* p* < 0.01] were significantly lower than those of non-pregnant participants even after adjusting for age, weight, and parity (Table 2).

## Discussion

In this study, the FVC, FEV1, and PEF values of pregnant and non-pregnant participants were lower than those predicted by their age and height. Also, pregnant participants' FVC, FEV1, and PEF values were lower than those of non-pregnant participants.

We report spirometry test values in women, which are comparable to values reported from other studies done in Tanzania [27], Rwanda [28], and Mozambique [29]. Our values were slightly higher than other African studies because of the age difference, as the mean age of non-pregnant participants was less than 30 years in this study, while it was more than 35 in the others. But these values are lower than the mean reported in Brazilians [44], Europeans and Australians [45], Asians [46], and Scandinavians [47] except for FEV1/FVC. Lower values have been reported among people of African decency and could not be explained by anthropometrics and skin color differences alone [5, 48]. A portion of this could be due to lower seating height and socioeconomic status, which relate to lower values. Yet, the values are normal since the prognosis has not been different [49–52]. We did not administer a bronchodilator before spirometry like in several other studies. This could have contributed to the lower spirometry values.

We noted a phasic relationship between age and spirometry test values of non-pregnant participants. The peak age for FVC, FEV1 and PEF was earlier, with lower values in pregnant participants. The spirometry test values are known to increase with age and then peak around 25 years before starting to decline [5, 47, 53, 54]. It may be a part of the aging process. After peak age, pulmonary elastic recoil decreases due to progressive loss of lung tissue elasticity and an increase in chest wall stiffness resulting in the decline of lung function [55–59]. Also, it could be partly due to a decrease in spirometry performance with aging. Age has been essential in spirometry test values predicting equations.

Similar to previous studies, the FVC, FEV1, and PEF of pregnant and non-pregnant participants increased with height [27, 28, 47]. Height has been essential in spirometry prediction equations as age [3, 27, 53, 60–65]. However, FVC% and FEV1% decreased as height increased. This could mean that as height increased, participants were more likely to have lower FVC and FEV1 values than expected. It could also be a reference equation over predicting expected values. Reference values have been reported to over-predict the spirometry test values in different populations [60, 61, 64] even when derived from a closely related population [65]. In line with other studies [47], there was no significant effect between height and FEV1/FVC. This could be due to the equal impact of height on FEV1 and FVC.

FVC, FEV1, and PEF of pregnant participants increased with weight, peaked at 61–70 kg, then decreased, but it was not statistically significant as has been reported by other studies [27, 28, 66, 67]. It could be because weight and BMI are not specific to the distribution of body composition, while fats in hips, thighs, gluteal regions, and breasts are less likely to affect lungs, diaphragm, and chest wall mechanics [46]. Other studies revealed a negative effect of the increasing waist-to-hip ratio (WHR) and weight gain on FEV1 and FVC [68, 69]. While this study was limited to FVC, FEV1, and PEF, other studies have found an inverse relationship between increasing BMI and vital capacity, total lung capacity, and functional residual capacity [70, 71].

The mean FVC, FVC% FEV1%, PEF, and PEF% were higher in parous than nulliparous, and the first birth showed the greatest effect. Despite that, only FVC% and FEV1% were statistically significantly related to parity in non-pregnant participants, and the relationship disappeared after adjusting for age, height, and weight, similar to another study [44]. Other researchers found a significantly adjusted positive effect of parity on spirometry test values [54, 67]. It has been postulated that the hormonal effects of pregnancy to compensate for mechanical changes and maintain lung function persist even after the uterus has returned to its small size [67, 72]. Similar to the other studies [44], the median FEV1/FVC ratio was lower in parous than nulliparous in pregnant and non-pregnant participants. Still, it was statistically significant only in pregnant participants after adjusting for age, height, and weight. This could be due to disproportionate changes between FVC and FEV1.

The spirometry test values decreased as the gestation age advanced, as in previous studies [44, 73, 74]. The decrease has been attributed to the limited maternal effort as gestation advances due to increased maternal weight, uterine enlargement, and a degree of pulmonary edema [75]. The spirometry test values have been observed to remain within the normal limits in other studies [18, 19]. But these studies focused on whether values were normal compared to a known range. Our analysis compared absolute values and their % of predicted values of pregnant participants at different gestational periods. Other studies have reported values that increased during pregnancy and persisted to the postpartum period [32, 66, 67].

FVC, FEV1, and PEF values were significantly lower than values predicted by age and height, as in another study done in Tanzania [76]. These findings could suggest that the reference equations have over-predicted expected values. The study involving young men in Tanzania found that reference equations developed from non-African populations overpredicted measurements of black Africans [76], as in other studies [60, 61, 64].

Compared to non-pregnant participants, the values of pregnant participants were significantly lower even after adjusting for age, weight, and parity. It could be explained by ribcage and volume displacement long known to occur during pregnancy [13, 15, 19, 77]. However, Le Merre et al. discussed that changes during pregnancy would not cause significant respiratory functional changes since hormonal factors balance the mechanical effects [78]. Unlike other studies that compared pregnancy values against the established normal range, this study compared the values of pregnant participants against those of non-pregnant participants.

Our study was not without limitations. Non-pregnant healthy women were more likely to hesitate to participate in the study as they would feel a lack of need for tests. Only pregnant women who booked their first visit in their first trimester were included. Pregnant participants were obtained by random sampling, while non-pregnant participants were obtained consecutively. Also, many potential participants hesitated to participate, worrying that they were being tested for Coronavirus. These factors could have influenced the nature of the participants who participated in this study and limited our ability to match the characteristics of pregnant participants. Our study was limited to spirometry; therefore, it could not explain other observations which would be well explained by other lung function tests, such as measuring static lung volumes. Also, we did not quantify hormonal effects on the spirometry profile by hormonal assay.

## Conclusion

Spirometry test values of pregnant women decrease as gestational age advances, and they are lower than profiles predicted by their age and height if they were not pregnant. Pregnant women's spirometry profiles are lower than those obtained from non-pregnant women. Pregnant and non-pregnant African women's spirometry profiles vary according to age, height, and parity. Weight or BMI does not affect the spirometry profiles of pregnant and non-pregnant women.

## Recommendation

Interpreting the spirometry test values of pregnant women using references obtained from non-pregnant women may be inappropriate. Future studies should evaluate the appropriateness of predicting spirometry values of pregnant women using reference equations derived from non-pregnant women. We recommend considering non-linear models when predicting the expected values of young women. Weight and BMI may not be suitable for studying the effect of body composition on spirometry profile; hence other measures should be considered.

## Data Availability

The datasets used during the current study are available from the corresponding author upon reasonable request.

## Abbreviations

ATS: American Thoracic Society
BMI: Body mass index
BMJ: British Medical Journal
CI: Confidence interval
Cm: Centimeter
ERS: European Respiratory Society
FEV1: Forced expiratory volume in 1 second
FVC: Forced vital capacity
Kg: Kilogram
L: Liter
ml: Milliliter
MUHAS: Muhimbili University of Health and Allied Sciences
NHANES: National Health and Nutrition Examination Survey
Non-preg: Non-pregnant
PEF: Peak expiratory flow
SD: Standard deviation
SEM: Standard error of the mean
SOP: Standard operating procedures
